# Use of Canonical Single Nucleotide Polymorphism (CanSNPs) to characterize *Bacillus anthracis* outbreak strains in Zambia between 1990 and 2014

**DOI:** 10.7717/peerj.5270

**Published:** 2018-07-26

**Authors:** Antonio Fasanella, Luigina Serrecchia, Alexandra Chiaverini, Giuliano Garofolo, Geoffrey M. Muuka, Lucas Mwambazi

**Affiliations:** 1 Anthrax Reference Institute of Italy, Istituto Zooprofilattico Sperimentale della Puglia e della Basilicata, Foggia, Italy; 2 Istituto Zooprofilattico Sperimentale dell’Abruzzo e del Molise “G. Caporale”, Teramo, Italy; 3 Bacteriology Section, Ministry of Fisheries and Livestock, Veterinary Services Department, Central Veterinary Research Institute, Lusaka, Zambia

**Keywords:** Anthrax, *Bacillus anthracis*, CanSNPs, Zambia, Outbreaks

## Abstract

Anthrax caused by *Bacillus anthracis* is an old and neglected zoonosis that continues to raise concerns in Southern Africa. In this study, twenty (20) slides with suspected isolates of *B. anthracis* from anthrax cases between 1990 and 2014 and two (2) from that of a vaccine strain were analysed using MLVA with 15 VNTRs and CanSNPs test. The results from the CanSNPs indicate that all anthrax outbreaks in Zambia between 1990 and 2014 were caused by the lineage A.Br.005/006 of the clade A. This indicates a common ancestral origin of the *B. anthracis* circulating in the country. This data has described several environmental, wildlife, livestock and human cases that occurred in a 24 year period, from the major areas where anthrax is endemic. The molecular characterization of isolates from anthrax outbreaks in Zambia has revealed a genetic structure in agreement with previous studies from neighbouring countries. Further studies are needed to elucidate how to better manage anthrax outbreaks and define the risk maps of Zambia.

## Introduction

*Bacillus anthracis*, the causative agent of anthrax an old and neglected fatal zoonotic disease, is a multi-host pathogen affecting human, livestock and wildlife populations. The disease has a worldwide distribution although it continues to pose more serious public health and socio-economic threat in several developing Asian and African countries (Dixon et al., 1999). Global dispersal of spores via commodities has been prevalent, such that there are currently endemic anthrax foci on all continents except Antarctica (http://www.vetmed.lsu.edu/ whocc/).

Anthrax is still endemic in Africa, with severe outbreaks causing significant losses (Prins & Weyerhaeuser, 1987; Shiferaw et al., 2002; Siamudaala et al., 2006; Clegg et al., 2007; Wafula, Patrick & Charles, 2008). Occasionally, these outbreaks occur in non-endemic areas (Turnbull & World Health Organization (WHO), 1998; Lewerin et al., 2010). In Zambia, outbreaks of this disease have occasionally been reported from different parts of the country and in the last decades, the number of anthrax cases has been increasing (Turnbull, Hugh-Jones & Cosivi, 1999; Tuchili et al., 1993). Several reports showed that anthrax outbreaks (Munang’andu et al., 1996) are higher in the Western Province and Luangwa valley (Turnbull, Hugh-Jones & Cosivi, 1999; Tuchili et al., 1993) than the rest of the country.

*B. anthracis* is a Gram positive, capsulated, and spore-forming bacterium. The spores are very robust and can survive in suitable soil for several decades. In the Kruger National Park (Africa) *B. anthracis* spores have been isolated from animal bones estimated to be about 200 years old (Smith et al., 2000). The ability of *B. anthracis* spores to survive outside the body is key for the ecology and evolution of this pathogen (Higgins, 1916). The endemicity of *B. anthracis* in some areas has been associated with calcium rich and neutral-to-alkaline soils (Van Ness, 1971; Dragon & Rennie, 1995; Blackburn et al., 2007), although strain differences exist in soil chemistry preferences (Smith et al., 2000). Strain differences, in fact, may also govern spread in anthrax epidemics (Blackburn et al., 2007; Garofolo et al., 2010). *B. anthracis* virulence is due to the anthrax toxin and anti-phagocytic capsule. Genes encoding the anthrax toxin are located on the pXO1 plasmid (Uchida et al., 1993; Okinaka et al., 1999) and those encoding the capsule are located on the pXO2 plasmid (Keppie, Harris-Smith & Smith, 1963; Makino et al., 1989). Anthrax toxin consists of three proteins namely: protective antigen (PA), edema factor (EF) and lethal factor (LF).

*B. anthracis* is a relatively homogeneous bacteria species which may be due to the long periods its endospores spend being dormant during its lifecycle thus does not have the opportunity to accumulate DNA mutations (Van Ert et al., 2007a; Van Ert et al., 2007b). Although this low genetic variability has prevented traditional molecular typing in differentiating *B. anthracis* isolates, two classes of genetic markers, the variable-number tandem repeats (VNTRs) and the canonical SNPs (canSNP), have proved to be highly effective (Thierry et al., 2014). The high discriminatory power of Multi locus VNTR analysis (MLVA) has allowed the identification of the little genetic diversity differentiating *B. anthracis* in two major lineages (A and B). However, the whole genome single nucleotide polymorphism (SNP) analysis, have greatly enhanced genotyping and phylogenetic analyses among *B. anthracis* isolates (Keim et al., 2009; Pilo & Frey, 2011). These genetic markers have allowed better understanding of how isolates fit into regional and global phylogeographic patterns (Pearson et al., 2004; Van Ert et al., 2007a; Van Ert et al., 2007b; Simonson et al., 2009). *B. anthracis* is divided into three (3) clades, namely A, B and C with further subdivisions into 13 canonical lineages and genetic groups (Van Ert et al., 2007a; Van Ert et al., 2007b; Marston et al., 2011). Clade A has the broadest distribution in the world with the A-lineage isolates recovered from five continents. Strains within this group account for 85% of worldwide anthrax outbreaks (Keim et al., 2009; Van Ert et al., 2007a; Van Ert et al., 2007b).

In this study, we compared and analysed isolates of *B. anthracis* from various epidemics in Zambia from 1990 to 2014. Anthrax outbreaks have been recorded in Zambia since 1989 (Turnbull et al., 1991; Tuchili et al., 1993; Siamudaala et al., 2006). No previous studies have been conducted to demonstrate the relationship between the various anthrax isolates from various epidemics in Zambia although Ohnishi et al. (2011) showed lack of strain differences from *B. anthracis* isolates from a single epidemic using MLVA genotyping comprising 21 VNTRs. The study used canSNPs and MLVA to analyse genetic relationships of anthrax in Zambia and to identify their possible global epidemiological correlations. The various sources of the isolates from these epidemics include soil, wildlife, livestock and humans.

## Methods and Materials

### Samples and DNA extraction

Twenty-two (22) slides with suspected isolates of *B. anthracis* from anthrax cases and one (1) slide from that of vaccine strain Sterne strain 34F_2_ were analysed. Each slide was incubated in deionized water for 24 h at room temperature. After 24 h, a scalpel was used to scrape off the material from the slide into a 2 ml Eppendorf tube. An aliquot of this material was then been treated to extract DNA by DNeasy® Blood & Tissue Kit (Qiagen, Hilden, Germany), according to the manufacturer’s instructions; while the other aliquot was used to isolate *B. anthracis* using the semi-selective medium trimethoprim sulfamethoxazole methanol polymyxin (TSMP) agar. From the suspect colonies, after heat inactivation, microbial DNA was extracted using the previous mentioned kit.

### Genotyping using canSNPs and MLVA-15

The *B. anthracis* identification was performed using specific PCR assays (Fasanella et al., 2001). The canSNP profiles were recorded, using 13 TaqManTM-Minor Groove Binding (MGB) allelic discrimination assays with primers and probes as described by Van Ert et al. (2007a) and Van Ert et al. (2007b) and the results compared to the 12 recognized worldwide sub-lineages and sub-groups (Van Ert et al., 2007a; Van Ert et al., 2007b).

The MLVA analysis with 15 VNTR primers used the method described by Van Ert et al. (2007a) and Van Ert et al. (2007b). Briefly, the MLVA PCRs were performed in four multiplex reactions in a final volume of 15 µl. Reaction mixture contained 1× Roche PCR reaction buffer, 1 U of Taq DNA polymerase (Roche), dNTPs (0.2 mM each), the appropriate concentrations of each primer as reported in Lista et al. (2006). The thermocycling conditions were 96 °C for 3 min; 36 cycles of 95 °C for 20 s, 60 °C for 30 s, and 72 °C for 1 min; and finally, 72 °C for 10 min. The amplicons obtained were diluted at the optimal concentration and were subjected to capillary electrophoresis.

We paired original MLVA data from our study with data from the MLVA international database (http://microbesgenotyping.i2bc.paris-saclay.fr/) to measure the VNTRs discrimination capacity and to carry on a global clustering. For each VNTR locus the Simpson’s diversity indices (SDIs) were calculated using the VNTR diversity and confidence extractor software (V-DICE) (http://www.hpa-bioinfotools.org.uk/cgi-bin/DICI/DICI.pl).

Minimum spanning trees were carried out using goeBURST algorithm, implemented in Phyloviz 2.0 software (Gonçalves et al., 2016).

## Results

We isolated *B. anthracis* from three out of 22 microbiological slides. The PCR assays carried out on the DNAs extracted directly from the slides found 11 samples positive for *B. anthracis* (Table 1).

**Table 1:** *B. anthracis* isolates used in the study. The alleles of the VNTRs are expressed in base pairs.

Slide-id	Source	Can SNPs	MLVA15 genotype	pXO1	vrrb2	vrrb1	vrra	vrrc1	pXO2	vrrc2	cg3	vntr19	vntr16	vntr32	Vntr12	Vntr17	Vntr 35	Vntr 23
6	DNA	A Br.005/006	A	121	154	223	306	620	131	522	153	93	271	440	110	381	109	193
7	Strain	A Br.005/006	A	121	154	223	306	620	131	522	153	93	271	440	110	381	109	193
8	DNA	A Br.005/006	A	121	154	223	306	620	131	522	153	93	271	440	110	381	109	193
11	DNA	A Br.005/006	A	121	154	223	306	620	131	522	153	93	271	440	110	381	109	193
13	DNA	A Br.005/006	B	121	154	223	306	620	131	522	153	93	271	440	110	390	109	193
14	Strain	A Br.005/006	A	121	154	223	306	620	131	522	153	93	271	440	110	381	109	193
15	DNA	A Br.005/006	B	121	154	223	306	620	131	522	153	93	271	440	110	390	109	193
17	Strain	A Br.005/006	A	121	154	223	306	620	131	522	153	93	271	440	110	381	109	193
18	DNA	A Br.005/006	B	121	154	223	306	620	131	522	153	93	271	440	110	390	109	193
19	DNA	A Br.005/006	B	121	154	223	306	620	131	522	153	93	271	440	110	390	109	193
Strain vaccine	DNA	A Br.001/002	C	127	154	223	306		–	522	153	90	–	563	110		116	182

Next, canSNP analysis demonstrated that all strains belonged to the Ancient A group A.Br.005/006 of Zambia as expected and previously established by Birdsell et al. (2012). The Sterne strains belongs to A.Br.001/002.

The MLVA analysis with 15 VNTRs panel showed two different genotypes (Table 1). The genotype A was retrieved in four slides, the genotype B in seven, while genotype C was detected from the Sterne vaccine strain. All the live *B. anthracis* showed the same genotype A. The genetic variation between the genotypes from the wild strains was in the locus VNTR17, which showed two alleles with a difference of only one repeat unit. This little genetic differentiation suggest the presence of a spore resilience or high sporulation efficacy in the original sampled specimens.

The discriminatory power of each VNTRs was estimated by the number of alleles detected and the allele diversity, and was reported in Table 2. The SDIs ranged from 0.095 (locus vrrb1) to 0.776 (locus pXO1); the hyper variable loci were the Bavntr17, pXO1, pXO2 and Bams01 with respectively 0.542, 0.776, 0.739 and 0.682 SDIs. In order to carry out a consistent global phylogenetic reconstruction we refined the MLVA panel to 11 markers discarding the four hyper-variable loci.

**Table 2:** The discriminatory indexes of the 15VNTRs with the relative confidence intervals, number of different alleles present in this sample set (*k*), and the fraction of samples that have the most frequent repeat number in this locus (Max(pi)).

Locus	Diversity index	Confidence interval	*k*	Max(pi)
vrra	0.169	0.134–0.204	7	0.91
vrrb1	0.095	0.067–0.124	6	0.951
vrrb2	0.102	0.073–0.131	5	0.947
vrrc1	0.408	0.369–0.448	10	0.753
vrrc2	0.429	0.400–0.457	5	0.704
cg3	0.341	0.308–0.374	3	0.783
pxo1	0.776	0.760–0.793	10	0.363
pxo2	0.739	0.724–0.753	11	0.365
Bams01	0.682	0.665–0.700	10	0.442
vntr12	0.099	0.071–0.128	5	0.948
vntr16	0.316	0.275–0.357	12	0.822
vntr17	0.542	0.519–0.566	9	0.584
vntr19	0.374	0.342–0.406	4	0.756
vntr23	0.233	0.196–0.271	5	0.871
vntr35	0.397	0.359–0.435	5	0.759

**Figure 1:**
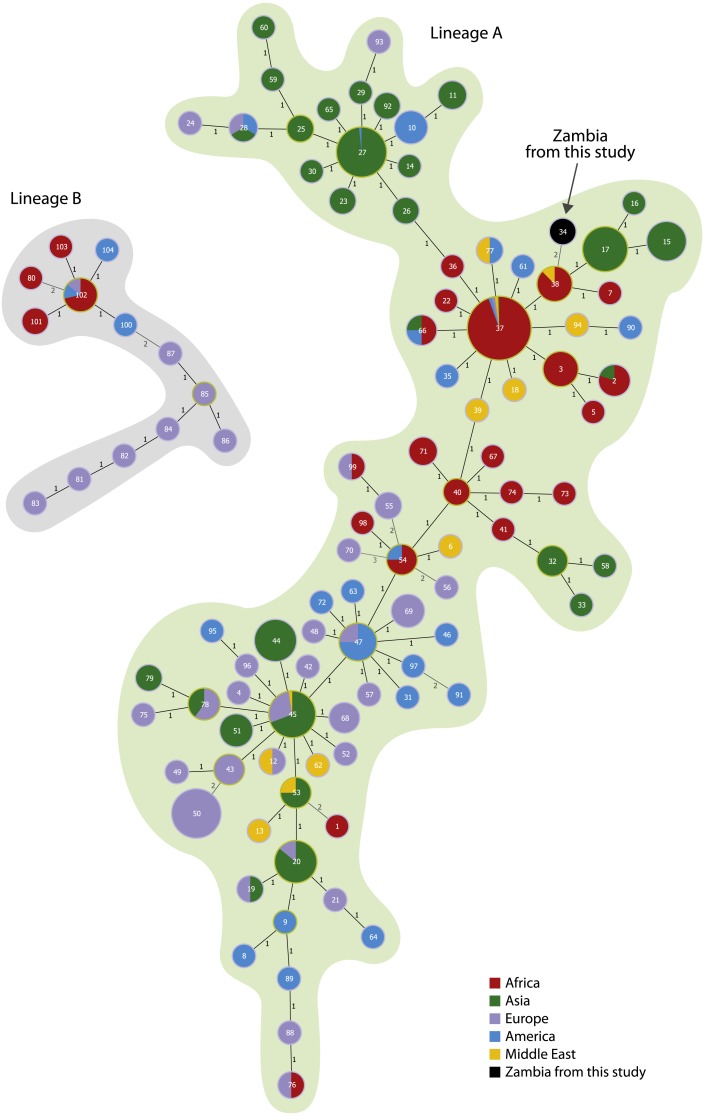
Minimum spanning tree on 799 isolates using the new MLVA-11 panel. Each circle coloured according to the continent source corresponds to one MLVA-11 genotype. The isolates from this study are coloured in black. The metadata from the isolates are reported in the Table S1.

**Figure 2:**
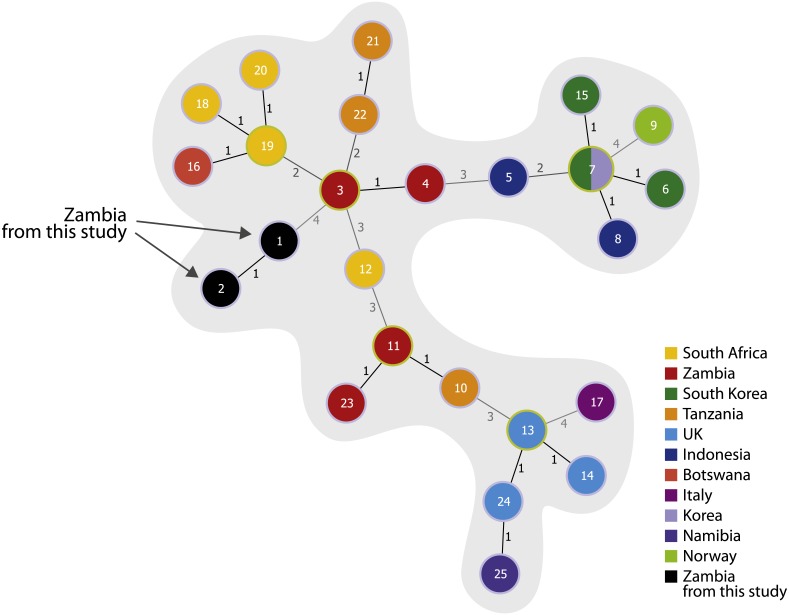
Minimum spanning tree on 35 isolates from the A.Br.005/006 lineage using the complete MLVA-15 panel. Each circle coloured according to the country source corresponds to one MLVA-15 genotype. The isolates from this study are coloured in black. The metadata from the isolates are reported in Table S2.

The minimum spanning tree with the new MLVA-11 panel split the global *B. anthracis* population into two large clonal complexes confirming the presence of two main lineages. The Zambian strains were found close to the Namibian strains (Fig. 1). The MST using the complete MLVA-15 panel was applied only for the group A.Br.005/006, finding the isolates from Zambia closely related with Southern African isolates (Fig. 2).

## Discussion & Conclusions

In the present study, it was possible to isolate live *B. anthracis* from slides that have been produced in the laboratory as a confirmatory assay of anthrax disease. The methylene blue stained slides were produced by heat fixation. Our observations demonstrate that this procedure is not suitable for complete killing of spores. Despite the resistance of this bacterium, it was demonstrated in the past by Jacotot & Virat (1954), isolated *B. anthracis* from slides cultured by Pasteur in 1870 and inoculated it in rats and induced the disease. This finding, confirms that spores are tough forms and slides taken from anthrax carcasses have to be treated as a potential hazard.

This is the first report from Zambia that classifies the anthrax isolates from outbreaks over a period of 24 years despite the high prevalence of the disease in this country. This study demonstrated that the *B. anthracis* strains involved in the outbreaks belong to the A.Br.005/006 group according to the categories described by Van Ert et al. (2007a) and Van Ert et al. (2007b), which states that the common lineages of *B.anthracis* in the Southern African region belong to either the A or B lineages. This agrees with earlier results from neighbouring countries of Zambia; for example, it was shown that Zimbabwe and South Africa that equally have reported high incidences of anthrax outbreaks in the past have both the A and B lineages (Smith et al., 2000; Van Ert et al., 2007a; Van Ert et al., 2007b). Our analysis showed a close genetic relatedness between *B. anthracis* from Zambia and Namibia, where most outbreaks were due to the A lineage (Beyer et al., 2012). This could be explained by the fact that Zambia and Namibia share common borders and the *B. anthracis* from the two countries could belong to the same ancestor.

The predominant A.Br.005/006 lineage in Zambia, which belongs to the world dominant A lineage linked to most outbreaks of anthrax, may indicate adaptive genetic mechanisms that have promoted the survival and propagation of this lineage in either the environment or hosts. The dispersal of this group to affect most areas of the country could be attributed to the other factors such as human behaviour, scavengers and climatic effects such as floods and droughts.

The group A.Br.005/006 is considered the ancient group; nevertheless its presence in Zambia as well the other Southern African countries demonstrate that the first selection of this group probably occurred in Africa. After that the lineage A started to spread all over the world with the TransEuroAsian group that is now ecologically established in Europe and the Middle East. Conversely, ABr.001/002, where the Sterne Vaccine isolate belongs, is believed to be a more recent group probably selected in Asia and reintroduced in Africa either through introduction of domesticated livestock from Asia or by modern trades within the Commonwealth countries.

This report successfully relates the phylogenetic connection of the Zambian *B. anthracis* strains in a global phylogeny. These data have described several environmental, wildlife, livestock and human cases that occurred in a 24 years period from the major areas where anthrax is endemic and have revealed the actual spread of anthrax in the country. In conclusion, the molecular characterization of isolates from anthrax outbreaks in Zambia has revealed a genetic structure in agreement with previous studies from neighbouring countries. Further studies are required to assess the management of anthrax outbreaks and define the risk maps of the disease in Zambia.

##  Supplemental Information

10.7717/peerj.5270/supp-1 Table S1 The metadata from the isolates in Fig. 1

10.7717/peerj.5270/supp-2 Table S2 The metadata from the isolates in Fig. 2

## Additional Information and Declarations

### Data Availability

The following information was supplied regarding data availability: The raw data are provided in the Supplemental Files.
